# Results and Exploratory Biomarker Analyses of a Phase II Study CHANGEABLE: Combination of PD‐1 Inhibitor and Niraparib in GErm‐Line‐mutAted Metastatic Breast Cancer

**DOI:** 10.1002/mco2.70684

**Published:** 2026-03-15

**Authors:** Jian Zhang, Yiqun Du, Yanchun Meng, Yizi Jin, Mingxi Lin, Xuchen Shao, Xiaojun Liu, Yuxin Mu, Yun Liu, Zhen Hu

**Affiliations:** ^1^ Phase I Unit Fudan University Shanghai Cancer Center Shanghai China; ^2^ Department of Oncology Shanghai Medical College Fudan University Shanghai China; ^3^ Department of Medical Oncology Fudan University Shanghai Cancer Center Shanghai China; ^4^ State Key Laboratory of Systems Medicine for Cancer, Shanghai Cancer Institute, Renji Hospital, Shanghai Jiao Tong University School of Medicine Shanghai China; ^5^ Department of Breast Surgery Fudan University Shanghai Cancer Center Shanghai China

**Keywords:** BRCA mutation, homologous recombination deficiency, metastatic breast cancer, niraparib, programmed cell death protein 1 (PD‐1) inhibitor

## Abstract

This phase II trial evaluated the efficacy and safety of combining niraparib with the PD‐1 inhibitor HX008 in patients with metastatic breast cancer who had germline DNA damage response (DDR) mutations. The study included 37 patients, divided into a primary cohort of HER2‐negative individuals with germline BRCA1/2 or PALB2 mutations (*n* = 29) and an exploratory cohort of patients who were either HER2‐negative with other DDR mutations, had brain metastases, or were HER2‐positive (*n* = 8). The main cohort achieved an objective response rate (ORR) of 76% and a disease control rate (DCR) of 97%, with a median progression‐free survival (PFS) of 7.3 months. The exploratory cohort had an ORR of 25% and a DCR of 75%, while patients with brain metastases showed a 40% ORR. Among treatment‐related adverse events of Grade 3 or higher, the most frequently observed were anemia (35.1%), thrombocytopenia (10.8%), and neutropenia (8.1%). No treatment‐related deaths were reported. Somatic XPO1 mutations correlated with better response. Somatic TP53 mutations significantly correlated with shorter PFS, while ASXL1 mutations correlated with longer PFS. This chemotherapy‐free regimen demonstrates promising efficacy and a tolerable safety profile in patients with metastatic breast cancer and germline DDR mutations, providing a novel therapeutic option for this patient population, even those with brain metastases.

## Graphical abstract

This phase II trial evaluated the efficacy and safety of niraparib combined with HX008 in metastatic breast cancer (MBC) patients with germline DNA damage response (DDR) gene mutations. This chemotherapy‐free regimen demonstrates promising efficacy and a tolerable safety profile in MBC patients with germline DDR mutations, providing a novel therapeutic option for this patient population, even those with brain metastases.

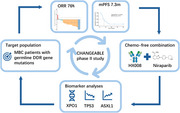

## 1 Introduction

Therapeutic strategies targeting homologous recombination deficiency (HRD) have shown great prospects in patients with breast cancer, particularly with the development of poly (ADP‐ribose) polymerase (PARP) inhibitors. HRD can be triggered by loss‐of‐function mutations or aberrant DNA methylation within the homologous recombination repair pathways, thereby driving genomic and chromosomal instability. Approximately 20% of breast cancer patients harbor HRD, and these individuals exhibit heightened sensitivity to PARP inhibitors [1, 2]. To date, several PARP inhibitors (olaparib, niraparib, talazoparib, and veliparib) have been approved globally for the treatment of ovarian and/or breast cancer. In the phase III OlympiAD study, olaparib showed significantly improved objective response rate (ORR, 59.9% vs. 28.8%) and progression‐free survival (PFS, 7.0 vs. 4.2 months, hazard ratio 0.58, *p* < 0.001) compared with standard chemotherapy in patients with germline BRCA1/2‐mutated, human epidermal growth factor receptor 2 (HER2)‐negative metastatic breast cancer [3]. Niraparib is an oral PARP1/2 inhibitor, and was first approved for the maintenance treatment of patients with platinum‐sensitive recurrent ovarian cancer. Clinical trials are also underway for the use of niraparib in breast cancer treatment [4, 5, 6].

Pucotenlimab (HX008) is a humanized immunoglobulin G4 (IgG4) monoclonal antibody targeting programmed cell death protein 1 (PD‐1), which selectively inhibits the interaction between PD‐1 and its ligands PD‐L1/PD‐L2. A phase I clinical trial has validated its safety profile in patients with advanced solid tumors [7]. Its clinical efficacy has also been demonstrated in the first‐line treatment of metastatic triple‐negative breast cancer when combined with gemcitabine and cisplatin, with ORR reaching 80.6% [8].

The synergistic potential of PARP inhibitors and immunotherapy is biologically plausible. PARP inhibition accumulates DNA damage, exposes tumor‐specific antigens, and activates the cyclic GMP‐AMP synthase/stimulator of interferon genes (cGAS‐STING) pathway. These effects are even more pronounced in the HR‐deficient breast cancer models [9, 10]. This cascade enhances tumor immunogenicity, increases tumor‐infiltrating lymphocytes (TILs) [11], and modulates immune synapses, while also upregulating PD‐L1 expression [12]. Anti‐PD‐1 antibodies can counteract this PD‐L1‐mediated immunosuppression, creating a complementary therapeutic effect [13].

Clinical trials have begun to validate this combination strategy for the treatment of breast cancer. The MEDIOLA phase I/II trial reported promising activity (12‐week ORR 63%, disease control rate [DCR] 80%) with olaparib plus durvalumab in germline BRCA‐mutated MBC [14], while the TOPACIO trial showed niraparib combined with pembrolizumab yielded an ORR of 21% in unselected advanced TNBC (47% in somatic BRCA‐mutated subsets) [6]. However, more effective regimens tailored to germline DNA damage response (DDR) gene mutations beyond BRCA1/2 are lacking, and companion predictive biomarkers are yet to be explored.

Breast cancer with brain metastases represents a particularly urgent clinical challenge, as these patients have limited treatment options and poor outcomes [15, 16, 17]. Conventional therapies often fail due to inadequate blood–brain barrier (BBB) penetration, but preclinical and clinical evidence indicate that both PARP inhibitors and immune checkpoint inhibitors can cross the BBB and exert intracranial antitumor activity. Niraparib has demonstrated efficacy in BRCA‐mutated intracranial TNBC models, while PD‐1 inhibitors have shown activity in brain metastases from various cancers [18, 19].

Given the unmet clinical needs and mechanistic rationales, the CHANGEABLE trial was designed to evaluate the efficacy, safety, and candidate predictive biomarkers of niraparib combined with HX008 among patients with metastatic breast cancer and germline DDR mutations and to explore whether this regimen could benefit patients with brain metastases. Our key findings demonstrate that this chemotherapy‐free combination yields robust antitumor activity and delivers meaningful clinical benefits even in patients with brain metastases. This study not only provides a new therapeutic strategy for this patient population but also offers actionable biomarkers to guide personalized treatment decisions.

## 2 Results

### 2.1 Study Patients

**FIGURE 1:**
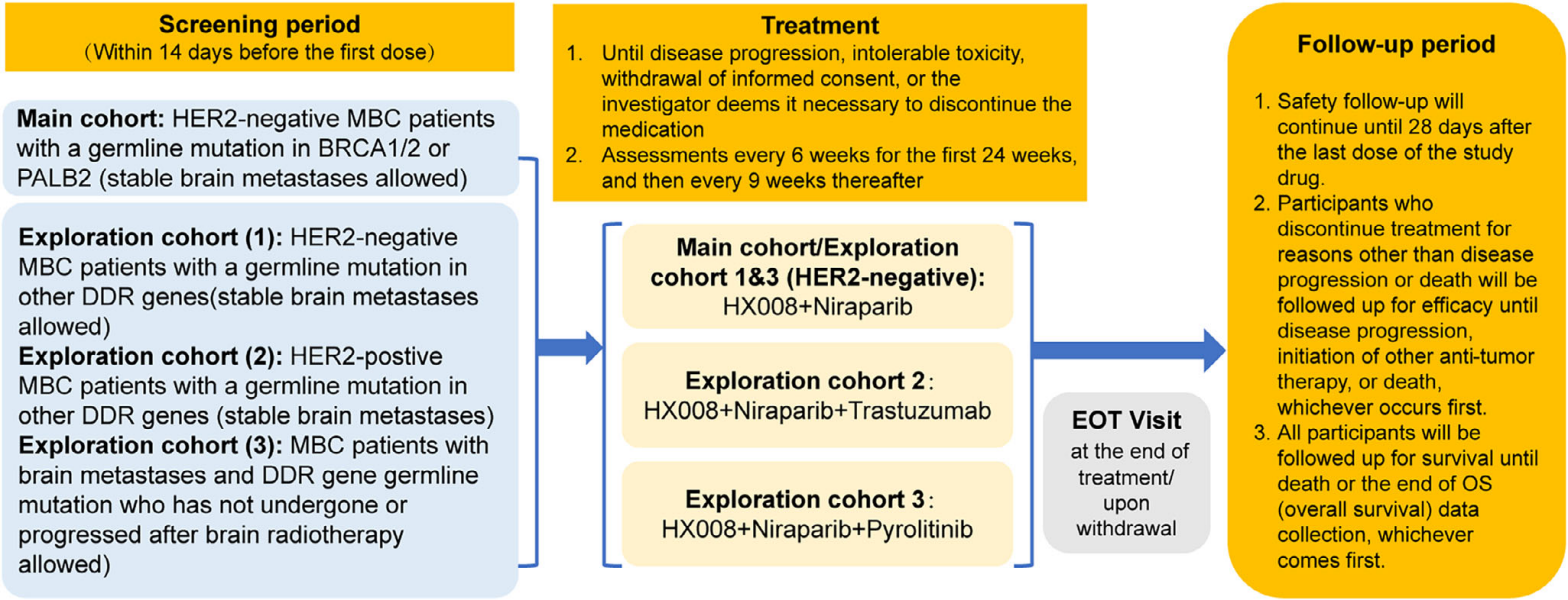
Study design and therapeutic schedule. EOT, end of treatment; DDR, DNA damage response; HER2, human epidermal growth factor receptor 2; MBC, metastatic breast cancer.

The study design and therapeutic schedule are illustrated in Figure 1. From September 2020 through February 2023, 37 patients were enrolled (including 29 patients in the main cohort and eight patients in the exploratory cohort). All patients received at least one cycle of treatment. The baseline characteristics of the patients are summarized in Table 1. Of patients enrolled, 46% had hormone receptor (HR)‐positive/HER2‐negative breast cancer, and 49% had triple‐negative breast cancer (TNBC). Eighty‐six percent of patients had visceral metastases, and five patients in the exploratory cohort had brain metastases. Prior therapies in the metastatic setting are summarized in Table 1, with additional details provided in Table S1. Seventy percent of patients received chemotherapy, and 38% patients had prior platinum treatment. In the main cohort, 52% of the patients had a mutation in BRCA1, and 45% had a mutation in BRCA2.

**TABLE 1:** Baseline characteristics.

Characteristics	All (*n* = 37), *n* (%)		Main cohort (*n* = 29), *n* (%)		Exploratory cohort (*n* = 8), *n* (%)	
Age, years, mean (range)	46 (27–70)		45 (27–66)		46 (36–70)	
Time since diagnosis, months, median (range)	40 (25–55)		37 (25–53)		46 (36–55)	
Subtype						
HR+/HER2‐	17	(46)	15	(52)	2	(25)
TNBC	18	(49)	14	(48)	4	(50)
HER2+	2	(5)	/	/	2	(25)
Visceral metastases	32	(86)	24	(83)	8	(100)
Brain metastases	5	(14)	/	/	5	(63)
No. of lines of prior chemotherapy in metastatic setting						
0	11	(30)	11	(38)	0	
1	18	(49)	13	(45)	5	(63)
2	5	(14)	3	(10)	2	(25)
3	3	(8)	2	(7)	1	(13)
Prior platinum in the metastatic setting	14	(38)	10	(34)	4	(50)
No. of lines of prior endocrine therapy in status metastatic setting						
0	21	(57)	15	(52)	6	(75)
1	4	(11)	4	(14)	0	
2	5	(14)	5	(17)	0	
≥3	7	(19)	5	(17)	2	(25)
PD‐L1 status^a^						
CPS ≥10	/	/	4	(14)	/	/
1≤ CPS <10	/	/	7	(24)	/	/
CPS <1	/	/	18	(62)	/	/
Germline mutations						
*BRCA1*	17	(46)	15	(52)	2	(25)
*BRCA2*	14	(38)	13	(45)	1	(13)
*PALB2*	3	(8)	1	(3)	2	(25)
*CHEK2*	3	(8)	/		3	(38)

Abbreviations: HER2, human epidermal growth factor receptor 2; HR, hormone receptor; TNBC, triple‐negative breast cancer.

^a^ PD‐L1 status was only detected in the main study cohort.

### 2.2 Efficacy

Data were analyzed up to March 2025. The median follow‐up was 27.9 months (range: 17.2–44.7 months). Efficacy by patient cohort is summarized in Table 2.

**TABLE 2:** Responses by patient cohort.

	Main cohort (*n* = 29)				Exploratory cohort (*n* = 8)	
**Response**	**All**	**gBRCA1/gBRCA2 mutations**	**gBRCA1 mutations**	**gBRCA2 mutations**	**All**	**Brain metastases**
Best response						
Complete response	3	3	3	0	0	
Partial response	19	19	8	11	2	2
Stable disease	6	5	3	2	4	2
Disease progression	1	1	1	0	2	1
ORR	76%	79%	73%	85%	25%	40%
DCR	97%	96%	93%	100%	75%	80%

Abbreviations: DCR, disease control rate; ORR, objective response rate.

**FIGURE 2:**
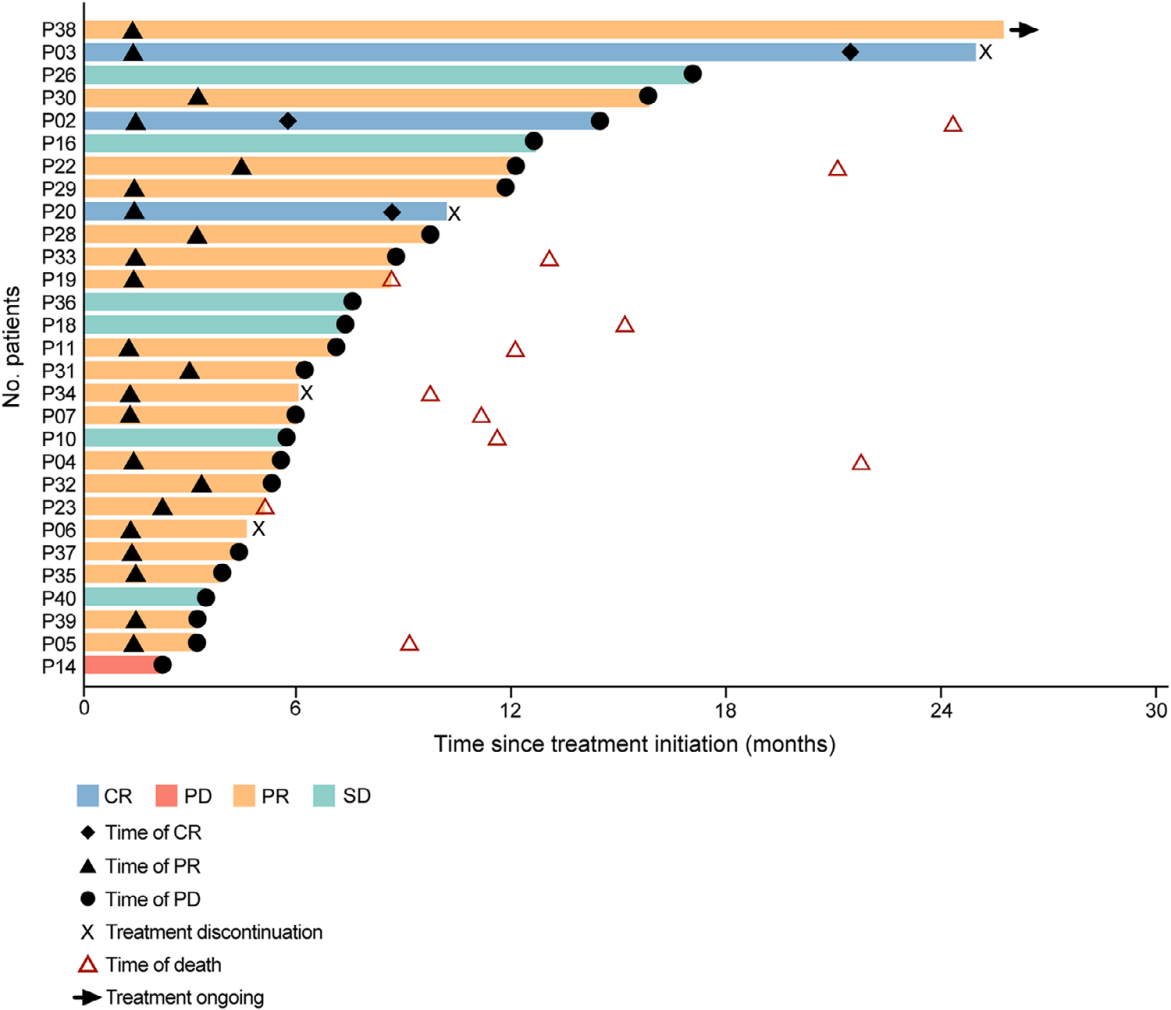
Treatment duration and best response in the main study cohort (*N* = 29). Each horizontal line represents an individual patient, with the length indicating the duration of treatment initiation to discontinuation (months). The best treatment response is indicated by the color coding. Key time points are marked: time of CR, time of PR, time of PD, time of death, and treatment discontinuation. CR, complete response; PR, partial response; SD, stable disease; PD, disease progression.

**FIGURE 3:**
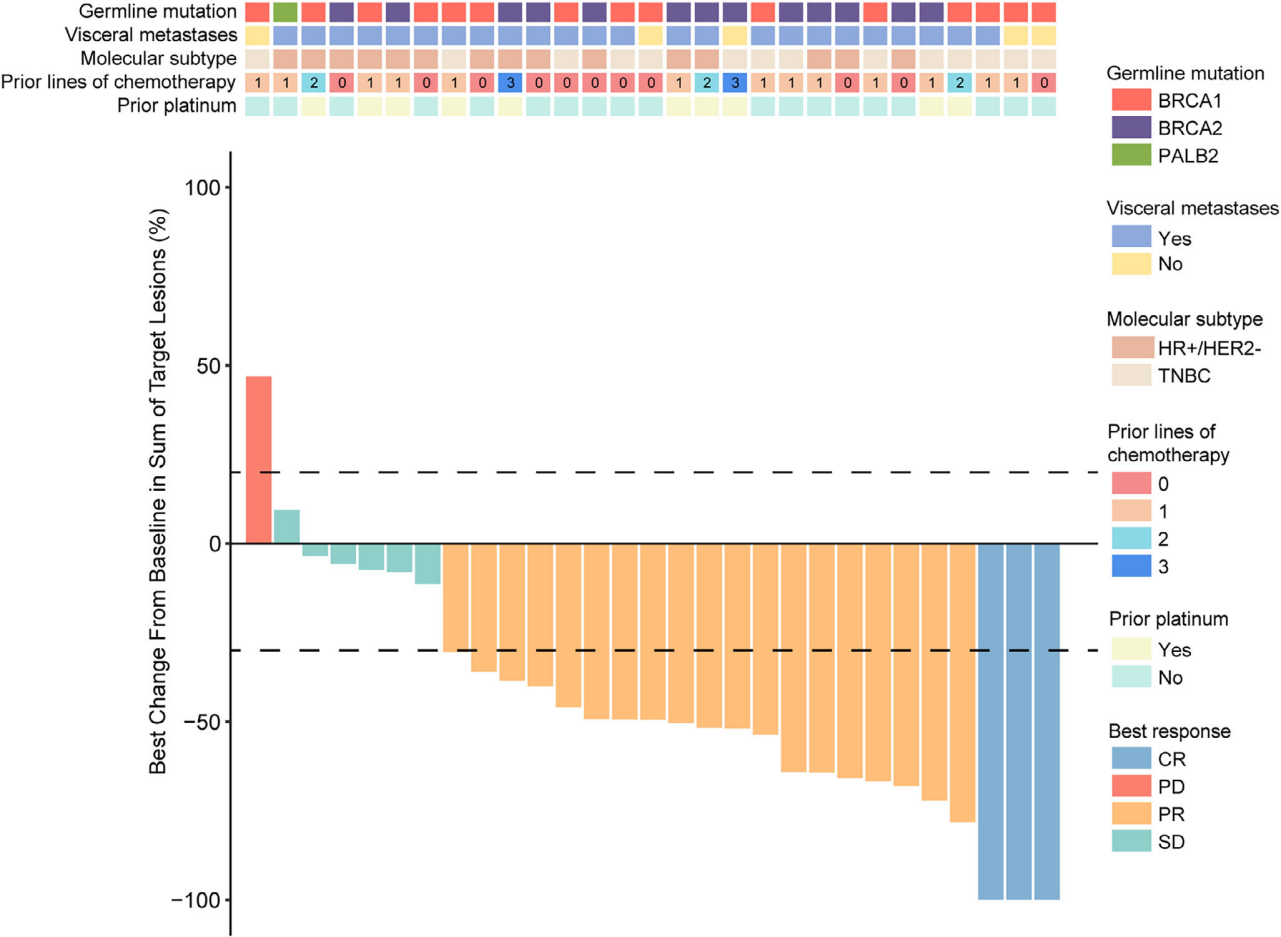
Best change from baseline in the main study cohort (*N* = 29). Each bar represents one participant, with the vertical axis indicating the percentage change in the sum of target lesions. Tumor assessments were performed in accordance with RECIST version 1.1 every 6 weeks until Week 24, then every 9 weeks thereafter. Patient characteristics are annotated by color coding and symbols above each bar: germline mutation status (BRCA1, BRCA2, PALB2), presence of visceral metastases (yes/no), molecular subtype (HR+/HER2−, TNBC), number of prior chemotherapy lines (0, 1, 2, 3), and prior platinum treatment (yes/no). Best responses are categorized as CR, PR, SD, or PD and labeled in different colors. HR, hormone receptor; HER2, human epidermal growth factor receptor 2; TNBC, triple negative breast cancer; CR, complete response; PR, partial response; SD, stable disease; PD, disease progression.

**FIGURE 4:**
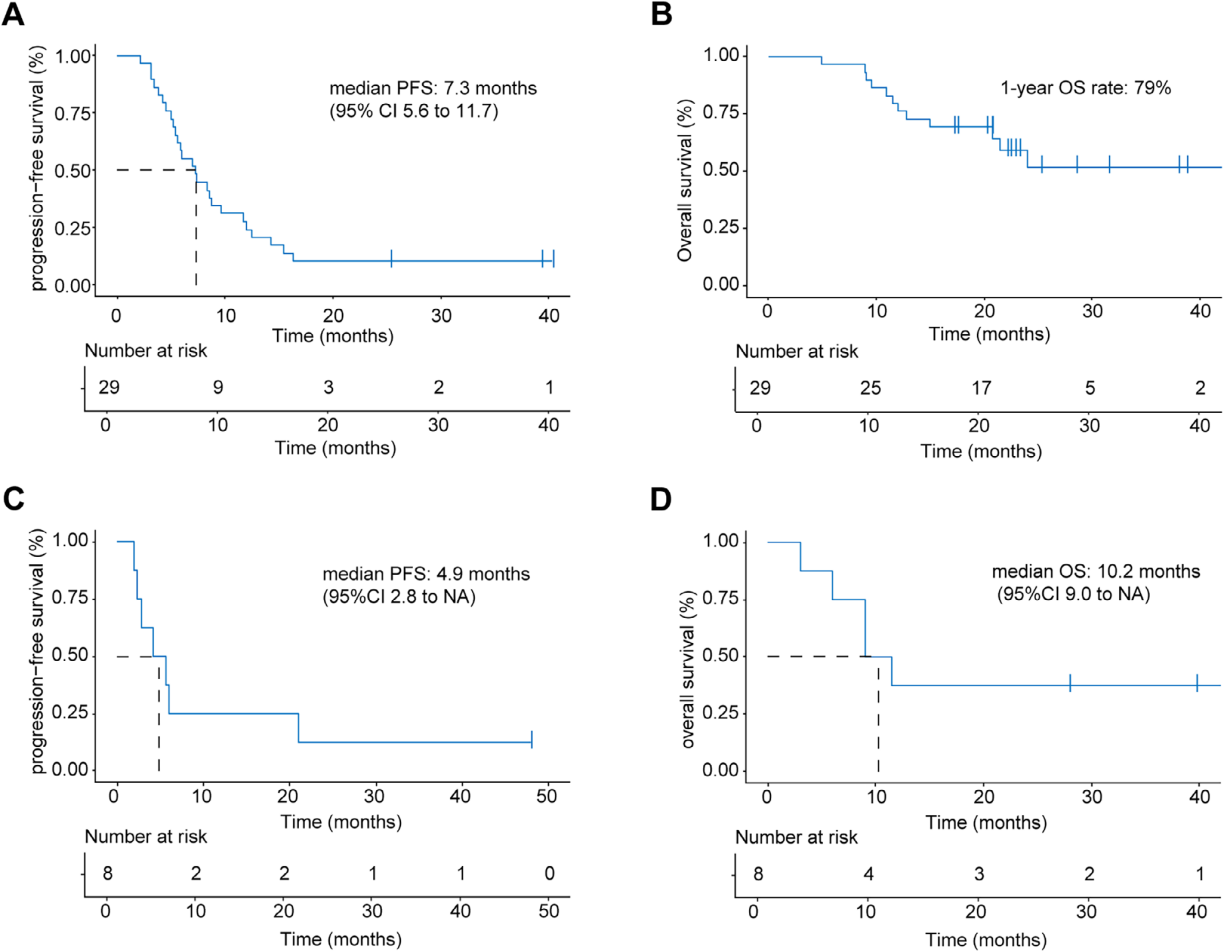
Efficacy analyses in the main study cohort and exploratory cohort. Kaplan–Meier survival curves for (A) PFS in the main study cohort; (B) OS in the main study cohort; (C) PFS in the exploratory cohort; (D) OS in the exploratory cohort. PFS, progression‐free survival; OS, overall survival.

The main cohort passed the initial stage. Treatment duration and best response in the main study cohort are summarized in Figure 2. The best change from baseline for each patient in the main cohort is illustrated in Figure 3. The ORR and the DCR were 76% (22/29) and 97% (28/29) in the main cohort, respectively; the median PFS was 7.3 months (95% confidence interval [CI]: 5.6–11.7, Figure 4A), and the 1‐year OS rate was 79% (Figure 4B). In the main cohort carrying germline BRCA1/2 mutations, the ORR was 79% (22/28), with a complete response observed in three patients. The disease control rate was 96% (27/28), and the median PFS was 7.4 months (95% CI: 5.4–12). The only patient with a germline PALB2 mutation had stable disease (SD) for 5.6 months. Subgroup analyses by clinical features demonstrated consistent efficacy across different subgroups (Table S2). Prior platinum exposure did not significantly impact clinical outcomes (platinum‐exposed vs. platinum‐naive: ORR 70% vs. 79%; DCR 100% vs. 95%; median PFS 6.0 vs. 8.5 months; Table S2).

For exploratory cohort, the ORR was 25% (2/8), with two patients having partial response (PR); the DCR was 75% (6/8); the median PFS was 4.9 months (95% CI: 2.8 to not reached [NR]) and the median overall survival (OS) was 10.2 months (95% CI: 9.0 to NR) (Table 2 and Figure 4C,D). In patients who have brain metastases (two germline BRCA1 carriers, one patient each with a germline mutation in BRCA2, PALB2, CHEK2), the ORR was 40% (2/5), with two patients having PR; the DCR was 80% (4/5); the median PFS was 4.1 months (95% CI: 2.8 to NA) and the median OS was 9.0 months (95% CI: 6.0 to NA). Two HER2‐negative patients with a germline CHEK2 mutation (without brain metastases) had SD for 21 and 2.7 months, respectively. The only patient with HER2‐positive breast cancer did not respond to the combination treatment and had a PFS of 2.3 months.

### 2.3 Safety

The combination therapy was generally well tolerated. The observed toxicity profile was consistent with previous studies (Table 3). The most common treatment‐related adverse events of any grade were anemia (22 [59.5%]), neutropenia (14 [37.8%]), lymphopenia (12 [32.4%]), and AST/ALT (aspartate aminotransferase/alanine aminotransferase) increased (9 [24.3%]). Among treatment‐related adverse events of Grade 3 or higher, the most frequently observed were anemia (13 [35.1%]), thrombocytopenia (4 [10.8%]), and neutropenia (3 [8.1%]). Patient data on dose reductions and treatment interruptions due to toxicity are detailed in Table S3. A total of 17 patients (45.9%) experienced a dose reduction of niraparib to 100 mg/qd during the treatment process. Among these, one patient had a dose delay of niraparib due to Grade 3 anemia, and reduced niraparib to 100 mg/qd in the subsequent treatment cycle. One patient discontinued niraparib because of long‐term Grade 4 thrombocytopenia. Immunotherapy‐related adverse events included pneumonitis (*n* = 1), myocarditis (*n* = 1), myositis (*n* = 1), hyperthyroidism (*n* = 1), and hepatitis (*n* = 1). Three patients (8.1%) had Grade 3 immune‐related adverse events. No treatment‐related deaths were observed.

**TABLE 3:** Treatment‐related adverse events.

Treatment‐related adverse event	All (*n* = 37), *n* (%)			
	Grade 1	Grade 2	Grade 3	Grade 4
Hematologic				
Anemia	8 (22)	1 (3)	12 (32)	1 (3)
Lymphopenia	2 (5)	8 (22)	2 (5)	0
Neutropenia	4 (11)	7 (19)	2 (5)	1 (3)
Thrombocytopenia	3 (8)	0	2 (5)	2 (5)
Immunotherapy‐related pneumonitis	0	0	1 (3)	0
Immunotherapy‐related myocarditis	0	0	1 (3)	0
Immunotherapy‐related myositis	0	1 (3)	0	0
Immunotherapy‐related hyperthyroidism	1 (3)	0	0	0
Immunotherapy‐related hepatitis	0	0	1 (3)	0
AST/ALT increased	6 (16)	2 (5)	1 (3)	0
Blood bilirubin increased	1 (3)	0	0	0
Creatinine increased	1 (3)	0	0	0
Cholesterol high	3 (8)	0	1 (3)	0
Hypertriglyceridemia	4 (11)	0	1 (3)	0
Hyperglycemia	3 (8)	0	1 (3)	0
Weight loss	0	3 (8)	0	0
Stomach pain	0	1 (3)	0	0
Anorexia	1 (3)	0	0	0
Fatigue	0	1 (3)	0	0
Middle ear inflammation	0	1 (3)	0	0
Hypothyroidism	1 (3)	2 (5)	0	0
Tachycardia	0	3 (8)	0	0
Interstitial pneumonia	0	1 (3)	0	0
Hypertension	0	1 (3)	0	0

Abbreviations: ALT, alanine aminotransferase; AST, aspartate aminotransferase.

### 2.4 Exploratory Analyses

**FIGURE 5:**
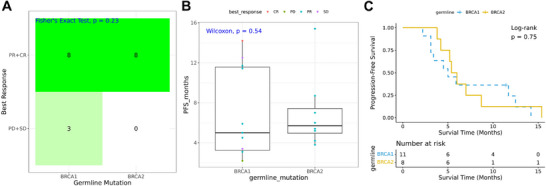
The correlation between germline BRCA1/2 mutation status and efficacy. (A) Fisher's exact test shows no significant differences in objective response; (B) the Wilcoxon test and (C) Kaplan–Meier curve show no significant differences in progression‐free survival (PFS) between patients with germline BRCA1 and BRCA2 mutations. CR, complete response; PR, partial response; SD, stable disease; PD, disease progression.

Biomarker profiling was performed using a 700+ gene panel of next‐generation sequencing on 52 specimens derived from 24 patients with germline BRCA1/2 mutations in the main cohort. No significant differences in objective response (CR+PR) (Fisher's exact test *p* = 0.23, Figure 5A) or PFS (Wilcoxon *p* = 0.54, Figure 5B; log‐rank *p* = 0.75, Figure 5C) were observed between patients with germline BRCA1 and BRCA2 mutations.

**FIGURE 6:**
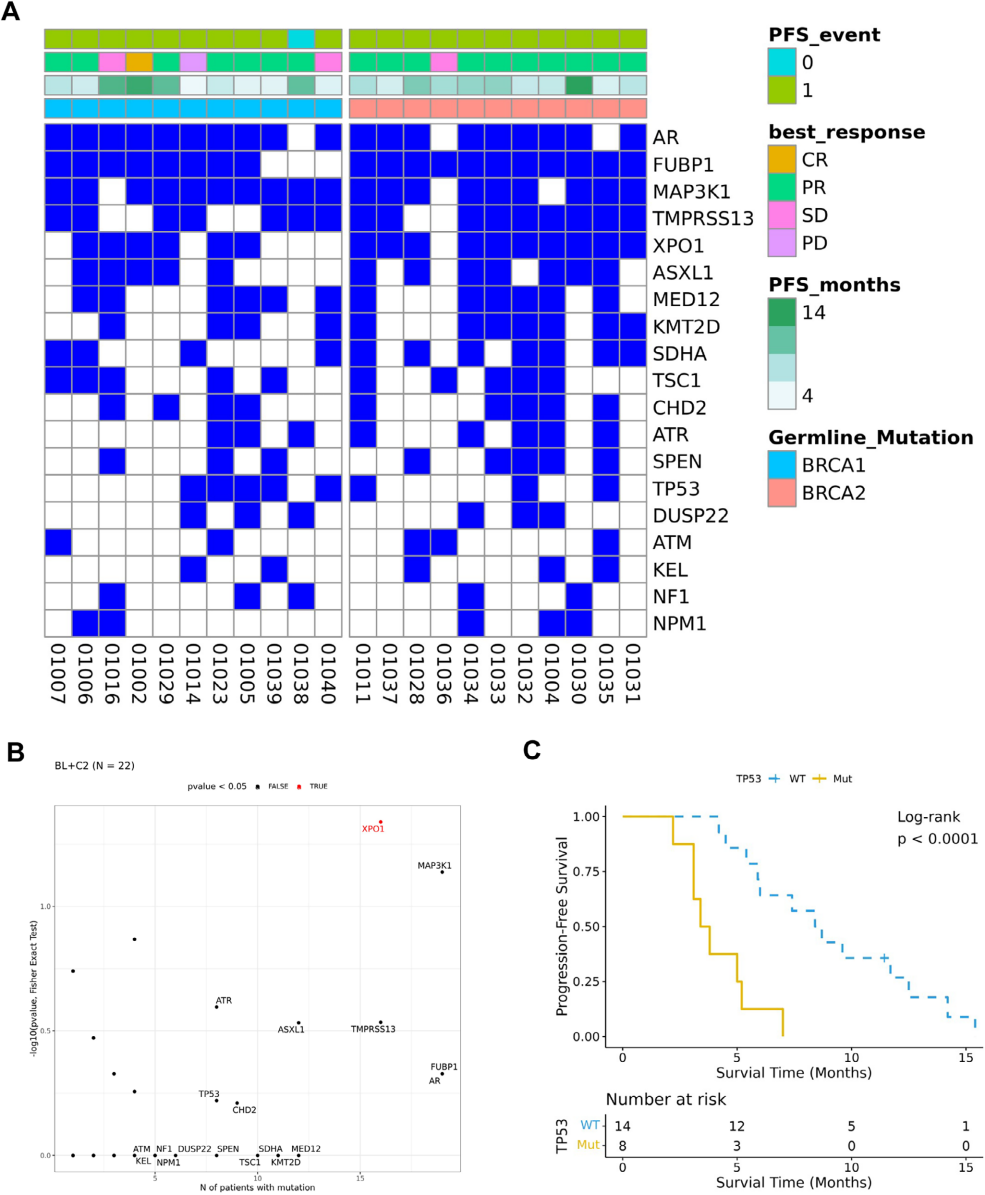
Biomarker analyses in the main study cohort. (A) Heatmap depicts somatically mutated genes in baseline and C2 samples. (B) Correlation between gene mutations and treatment response (CR+PR) by Fisher's exact test. (C) Kaplan–Meier curve of PFS stratified by TP53 somatic mutation status. CR, complete response; PR, partial response; SD, stable disease; PD, disease progression. PFS, progression‐free survival; WT, wild type; Mut, mutated.

Overview of somatically mutated genes (other than BRCA1/2) in samples collected at baseline and C2 is demonstrated in Figure 6A at the gene level and Figure S1 at the mutational level. Somatic mutations in XPO1 (16 patients [73%], 15/18 CR+PR, 1/4 SD+disease progression [PD]) showed significant correlation with response (*p* < 0.05, Figure 6B and Figure S2). Among the 19 somatic genes with mutations in at least five patients, mutations in TP53 were significantly correlated with shorter PFS (*p* < 0.0001, Figure 6C and Figure S3), while mutations in ASXL1 were significantly correlated with longer PFS (*p* < 0.05, Figure S3).

Four somatic mutations (AR_c.1418_1420del, AR_c.231_239dup, DUSP22_c.263+790G>A, DUSP22_c.263+768_263+769delGT) displayed a consistent tendency toward reduced frequency in PD samples. Conversely, a single somatic alteration (NF1_c.3198‐4dup) showed a persistent trend of increased prevalence in PD specimens (Table S4).

### 2.5 Post‐Protocol Treatment

Post‐protocol treatment for the main cohort is shown in Table S5. In the first‐line treatment after disease progression, 38% (11/29) of the patients were treated with chemotherapy (31% [9/29] platinum‐based), 21% (6/29) treated with antibody–drug conjugates (ADC), and only one patient used a CDK4/6 inhibitor combined with an aromatase inhibitor.

## 3 Discussion

In this open‐label, phase II clinical trial, we first reported the efficacy of niraparib combined with HX008, a PD‐1 inhibitor, in patients with metastatic breast cancer and germline DDR gene mutations, including patients with brain metastases. The ORR, DCR, and median PFS in the main cohort were 76%, 97%, and 7.3 months, respectively. The ORR and DCR for patients with DDR gene mutations and brain metastases reached 40% and 80%, respectively. Our study further demonstrated that somatic mutations in XPO1 were associated with a more favorable therapeutic response. In addition, TP53 mutations were significantly associated with shorter PFS, whereas ASXL1 mutations were significantly correlated with prolonged PFS.

The combination of PARP inhibitors and immune checkpoint inhibitors presents a novel and promising approach for treating breast cancer, especially in patients with HRD. Mechanically, PARP inhibition leads to the accumulation of DNA damage and exposure of tumor‐specific antigens, which activate the cGAS‐STING pathway and trigger an antitumor immune response. PARP inhibition was also reported to increase tumor‐infiltrating lymphocytes (TILs) and modulate the immune synapse. Olaparib and veliparib were found to enhance FAS and death receptor 5 expression on the cell surface and sensitize tumor cells to death receptor‐mediated apoptosis [20]. Of note, the activation of the STING pathway by DNA damage was also associated with enhanced PD‐L1 expression, which may partly explain the attenuated T‐cell cytotoxicity in DNA damage response‐deficient tumors [21, 22]. Therefore, anti‐PD‐1 monoclonal antibodies may compensate for this defect in PARP inhibitors.

Several clinical trials have investigated the efficacy and safety of combining PARP inhibitors with immunotherapies in breast cancer. In the MEDIOLA study, the combination of olaparib and durvalumab showed promising antitumor activity in patients with germline BRCA‐mutated metastatic breast cancer, with a 12‐week DCR of 80% and a 12‐week ORR of 63%. This combination was also investigated in the I‐SPY2 trial for patients with high‐risk HER2‐negative Stage II/III breast cancer in the neoadjuvant setting. The addition of durvalumab and olaparib to standard paclitaxel enhanced the pCR rates in all HER2‐negative patients [23]. The TOPACIO study evaluated another combination regimen of niraparib and pembrolizumab in unselected advanced or metastatic TNBC regardless of BRCA mutation status. The ORR was 21% (90% CI: 12%–33%) in all patients and 47% (90% CI: 24%–70%) in patients with somatic BRCA mutations. However, our study differs from prior trials in two key aspects: first, the proportion of patients with visceral metastases in our main cohort exceeded 80%, which is notably higher than in MEDIOLA (∼60%) and TOPACIO (not explicitly reported but likely lower given its unselected population) and far higher than in trials of single‐agent PARP inhibitors (OlympiAD [24]: ∼60%; EMBRACA [25]: ∼40%). This high proportion of visceral metastases reflects a more advanced and clinically challenging patient population, yet our combination regimen still achieved a superior ORR (76%) and DCR (97%) compared to these prior studies. Second, unlike MEDIOLA and TOPACIO, our trial included a dedicated subset of patients with brain metastases (*n* = 5), and demonstrated meaningful efficacy (ORR 40%, DCR 80%). These differences highlight the unique value of our regimen in real‐world clinical scenarios where patients often present with extensive disease and intracranial involvement.

The treatment of breast cancer with brain metastases remains a significant challenge to tackle. Both PARP inhibitors and immune checkpoint inhibitors have shown the potency to cross the BBB and exhibit antitumor activity within the brain. In preclinical models of TNBC, niraparib penetrates intracranial tumor tissues with impressive efficacy in BRCA‐mutant breast cancer [26]. In this study, patients with DDR gene mutations and brain metastases had an ORR of 40%, and two patients achieved PR. The median PFS and median OS of patients with brain metastases were 4.1 and 9.0 months, respectively. These results are particularly notable given that brain metastases are typically excluded from most clinical trials, and standard therapies for this subgroup offer limited benefit. By explicitly including these patients and demonstrating clinical activity, our study provides critical evidence that the niraparib–HX008 combination may serve as a viable therapeutic option for DDR‐mutated MBC patients with brain metastases.

Biomarker analyses suggested that XPO1 mutations correlated with better response to the combination of niraparib and HX008. A previous study reported that XPO1 inhibition synergized with PARP1 inhibition in small cell lung cancer through targeting nuclear transport of FOXO3a [27]. The co‐treatment with olaparib and selinexor, an XPO1 inhibitor, also exhibited antitumor activity in TNBC irrespective of BRCA1 mutation status. ASXL1 mutations were significantly correlated with longer PFS in this study. In a phase Ib basket trial, concurrent mutations in ATM and ASXL1 predicted sustained clinical responses to venadaparib, a novel PARP1/2 inhibitor, in patients with pancreatic cancer [28]. TP53 mutation showed prognostic value in this combination treatment. Clinical and experimental evidence showed that de novo TP53 mutations may be correlated with resistance to PARP inhibitors for BRCA‐mutated cancer [29]. Mechanistically, in tumor cells with BRCA1/2 mutations, TP53 mutations may further exacerbate the dysfunction of homologous recombination repair (HRR), leading to resistance to PARP inhibitors. Additionally, TP53 mutations may cause dysfunction of cell cycle checkpoints, allowing cells to proliferate without repairing DNA damage, thereby decreasing their sensitivity to PARP inhibitors. Meanwhile, the relationship between TP53 mutations and anti‐PD‐1 immunotherapy is more complex, showing controversial evidence and warranting further investigation [30, 31, 32]. Further studies are needed to confirm these findings in larger cohorts and explore underlying biological mechanisms. It is of note that PD‐L1 status is important for interpreting the efficacy. The ORR and DCR reached 76% and 97%, respectively, in the main study cohort, with most of the patients presenting a combined positive score (CPS) lower than 10. These data demonstrated the promising efficacy of this treatment strategy and highlighted its clinical value irrespective of PD‐L1 status.

Several limitations of this study should be acknowledged. First, as an open‐label, single‐arm phase II trial without a comparator arm, we cannot definitively confirm whether the addition of HX008 provides incremental benefit over niraparib monotherapy, nor can we directly compare its efficacy with standard‐of‐care regimens. Second, the sample size is relatively small, particularly in the exploratory cohort (*n* = 8) and the brain metastases subgroup (*n* = 5), which limits the statistical power to draw definitive conclusions about these populations. Third, we did not collect sequential tissue samples beyond baseline and Cycle 2, which prevents dynamic monitoring of genomic changes or tumor microenvironment shifts that may correlate with treatment response or resistance. These limitations underscore the need for future larger scale, randomized controlled trials to validate the efficacy of niraparib plus HX008 against standard therapies. Additionally, studies with extended follow‐up, larger sample sizes for subgroup analyses, and sequential sampling are warranted to confirm the biomarker findings in this patient population.

In conclusion, this phase II trial confirmed the robust clinical efficacy and manageable toxicity of a chemotherapy‐free regimen, niraparib combined with HX008, in MBC patients with germline DDR mutations, including patients with brain metastases. Our preliminary biomarker analyses may provide guidance for the selection of patients in the future. Further studies are warranted to validate the biomarkers and compare this combination therapy with the standard of care in larger, randomized cohorts.

## 4 Materials and Methods

### 4.1 Patient Eligibility

Eligible patients were 18–75 years old and had histologically confirmed MBC with at least one extracranial measurable lesion by RECIST 1.1 criteria. Patients had to have a germline pathogenic or likely pathogenic mutation in one of the listed DDR genes: BRCA1/2, PALB2, CHEK2, ATM, ATR, BAP1, BARD1, BLM, BRIP1, CHEK1, CDK12, FANCA, FANCC, FANCD2, FANCE, FANCF, FANCM, MRE11A, NBN, PTEN, RAD50, RAD51C, RAD51D, WRN. Progression on more than two previous lines of chemotherapy for metastatic disease was not allowed. Patients eligible for platinum‐based chemotherapy or a PARP1 inhibitor must demonstrate no disease progression during treatment or within 8 weeks following its cessation in the recurrent/metastatic setting, and must relapse within 12 months after completing neoadjuvant or adjuvant therapy. For those with hormone receptor‐positive, HER2‐negative disease, at least one line of endocrine therapy should have been administered, followed by progression to recurrent or metastatic disease, or relapse occurring during adjuvant endocrine treatment or within 1 year of its completion. Patients with stable central nervous system (CNS) metastases were eligible for enrollment. Additional eligibility criteria comprised an Eastern Cooperative Oncology Group (ECOG) performance status score of 0–1, sufficient organ function, and a life expectancy longer than 3 months.

### 4.2 Study Design

This investigation was an open‐label, single‐arm, phase II clinical trial. Eligible patients were assigned to two separate cohorts: the main cohort, consisting of HER2‐negative metastatic breast cancer (MBC) patients harboring germline BRCA1/2 or PALB2 mutations, and the exploratory cohort, comprising MBC patients with germline mutations in other DNA damage repair (DDR) genes, those presenting with brain metastases, or patients with HER2‐positive MBC. Patient recruitment in the main cohort was conducted in accordance with Simon's two‐stage design.

### 4.3 Drug Administration

Treatment for HER2‐negative MBC included niraparib (Zai Lab Ltd.) 200 mg orally once daily combined with intravenous HX008 (Taizhou Hanzhong Biomedical Co. Ltd.), a recombinant humanized anti‐PD‐1 monoclonal antibody, at 200 mg every 3 weeks. For HER2‐positive patients, trastuzumab was added (8 mg/kg intravenously in the initial cycle, 6 mg/kg intravenously every 3 weeks subsequently), or pyrotinib 400 mg orally once daily was administered for those with brain metastases. All treatments were continued until disease progression.

### 4.4 End Points and Assessments

The primary endpoint was objective response rate (ORR), defined as confirmed complete response (CR) and partial response (PR) by RECIST version 1.1 (4 weeks of confirmation for CR/PR are required). The secondary endpoints included disease control rate (DCR; i.e., confirmed CR or PR or stable disease [SD]), progression‐free survival (PFS), overall survival (OS), and toxicity. Tumor evaluations were conducted every 6 weeks up to Week 24, and every 9 weeks in subsequent follow‑up periods. Adverse events were graded using the National Cancer Institute Common Terminology Criteria for Adverse Events (NCI‐CTCAE), version 4.0.

Blood samples were collected at baseline, after completing two cycles of treatment (C2), and at disease progression (PD). Tumor samples (preferably from metastases) were also collected for DNA extraction. For exploratory biomarker profiling, next‐generation sequencing (NGS) was conducted on both tissue and circulating tumor DNA (ctDNA) using a targeted gene panel encompassing more than 700 genes (Homgen Cancer NBC 768 Panel).

### 4.5 Quantification and Statistical Analysis

Simon's Two‐Stage Minimax design was used for calculating the sample size of the main study cohort. The family‐wise error rate (FWER) was set at 0.1, and the Type II error rate (*β*) was set at 0.2. According to previous studies, the null response rate (*P*_0_) was set at 0.6, and the desirable response rate (*P*_1_) was set at 0.8. In the first stage, 11 patients would be enrolled. An interim analysis would be performed at the end of the first stage. If six or less patients achieve objective response, the study will be terminated. If seven or more patients achieve an objective response in the first stage, another 13 patients would be enrolled in the second stage. A final analysis would be made for all patients enrolled in the two stages (*n* = 24) at the end of the second stage. If ≤17 patients achieve objective response at the final analysis, the efficacy will be deemed lower than expected. If more than 18 patients among the 24 subjects achieve objective response, a further confirmatory study is recommended. The probability of early termination of the study at the interim analysis (null hypothesis *H*_0_: *P ≤ P*_0_) is 46.7%. In case of a 10% dropout rate, at least 27 patients would be enrolled in the main cohort. No sample size requirement was made for the exploratory cohort.

Demographics, baseline characteristics, and efficacy were evaluated using the full analysis set (FAS), defined as all patients who received at least one dose of study treatment and not excluded from the study for administrative reasons. Safety was evaluated in all patients who received at least one dose of study treatment and had safety assessment. The baseline characteristics of patients and safety profile were summarized descriptively. For time‐to‐event outcomes, Kaplan–Meier methods were used to calculate the median and corresponding two‐sided 95% CIs. The proportional hazards assumptions were tested using −ln[−ln(survival)] plots. Exploratory biomarker analyses were performed for ORR and PFS using Fisher's exact tests and log‐rank tests, respectively. The Bonferroni method was used to adjust for multiple comparisons. All statistics were performed using R software 4.4.3.

## Author Contributions

Conception and design: J.Z., Z.H., Y.L. Supervision of the study: J.Z., Z.H., Y.L. Project administration: J.Z., Y.‐Q.D., Y.‐C.M., Y.‐Z.J. Provision of study materials or patients: J.Z., Y.‐Q.D., Y.‐C.M., Z.H., Y.L. Recruitment and treatment of patients: J.Z., Y.‐Q.D., Y.‐C.M., X.‐J.L., Y.‐X.M, X.‐C.S. Collection and assembly of data: all authors. Data analysis and interpretation: J.Z., Y.‐Z.J., Y.‐Q.D., Y.‐C.M. Manuscript writing: Y.‐Z.J. Manuscript revision: J.Z., Y.‐Z.J., Y.‐Q.D., Y.‐C.M., M.‐X. L. Final approval of manuscript: all authors.

## Funding

This study was supported by the National Key Research and Development Program of China (Grant No. 2023YFF1205000); National Natural Science Foundation of China (Grant No. 82072915, 82373359, 82503430); Project of Shanghai Municipal Health Commission (Grant No. 202140397); CSCO Cancer Research Fund (Grant Nos. Y‐HR2020MS‐0298, Y‐pierrefabre202102‐0066); Chinese Young Breast Experts Research project (Grant No. CYBER‐2021‐001); and Beijing Science and Technology Innovation Medical Development Foundation Key Project (Grant No. KC2022‐ZZ‐0091‐6).

## Ethics Statement

This study was conducted in accordance with the International Ethical Guidelines for Biomedical Research Involving Human Subjects, the International Conference on Harmonization Guidelines for Good Clinical Practice, and the Declaration of Helsinki. The protocol was approved by the Fudan University Shanghai Cancer Center Institutional Review Board (SCCIRB) (Approval No. 2008222‐26‐2107A). This study has been registered on https://clinicaltrials.gov/; ID: NCT04508803. This study is reported following the Consolidated Standards of Reporting Trials (CONSORT) reporting guideline and Transparent Reporting of Evaluations with Nonrandomized Designs (TREND) reporting guideline. All patients provided written informed consent before enrollment.

## Conflicts of Interest

The authors declare no conflicts of interest.

## Supporting information



Figure S1. Overview of somatic mutations other than BRCA1/2 in baseline and C2 samples.
Figure S2. Correlation between somatic mutations and treatment response (CR+PR) (at the mutational level).
Figure S3. Prognostic significance of gene mutations in baseline and C2 samples.
Table S1. Summary of prior therapies in the metastatic setting.
Table S2. Subgroup analyses of efficacy by clinical features.
Table S3. Patient data on dose reductions and treatment interruptions due to toxicity.
Table S4. Change in mutations from baseline and C2 samples to PD samples.
Table S5. Post‐protocol treatment for the main cohort.

## Data Availability

Any additional information required to reanalyze the data reported in this paper is available from the primary corresponding author, Zhen Hu, upon reasonable request.
