# Supplementary material for: Results and Exploratory Biomarker Analyses of a Phase II Study CHANGEABLE: Combination of PD‐1 Inhibitor and Niraparib in GErm‐Line‐mutAted Metastatic Breast Cancer
Source: MedComm (2020). 2026 Mar 15;7(4):e70684. doi: 10.1002/mco2.70684 (PMC13042650; doi:10.1002/mco2.70684)
Supplement: Supplementary file 1 — Figure S1. Overview of somatic mutations other than BRCA1/2 in baseline and C2 samples. Figure S2. Correlation between somatic mutations and treatment response (CR+PR) (at the mutational level). Figure S3. Prognostic significance of gene mutations in baseline and C2 samples. Table S1. Summary of prior therapies in the metastatic setting. Table S2. Subgroup analyses of efficacy by clinical features. Table S3. Patient data on dose reductions and treatment interruptions due to toxicity. Table S4. Change in mutations from baseline and C2 samples to PD samples. Table S5. Post‐protocol treatment for the main cohort. [file MCO2-7-e70684-s001.docx]

**Supplementary Appendix**

**Table of Contents**

[**Figure S1. Overview of somatic mutations other than BRCA1/2 in baseline and C2 samples.** 2](#_Toc221558277)

[**Figure S2. Correlation between somatic mutations and treatment response (CR+PR) (at mutational level).** 3](#_Toc221558278)

[**Figure S3. Prognostic significance of gene mutations in baseline and C2 samples.** 4](#_Toc221558279)

[**Table S1. Summary of prior therapies in the metastatic setting.** 5](#_Toc221558280)

[**Table S2. Subgroup analyses of efficacy by clinical features.** 7](#_Toc221558281)

[**Table S3. Patient data on dose reductions and treatment interruptions due to toxicity.** 8](#_Toc221558282)

[**Table S4. Change in mutations from baseline and C2 samples to PD samples.** 10](#_Toc221558283)

[**Table S5. Post-protocol treatment for the main cohort.** 12](#_Toc221558284)

**Figure S1. Overview of somatic mutations other than BRCA1/2 in baseline and C2 samples.**


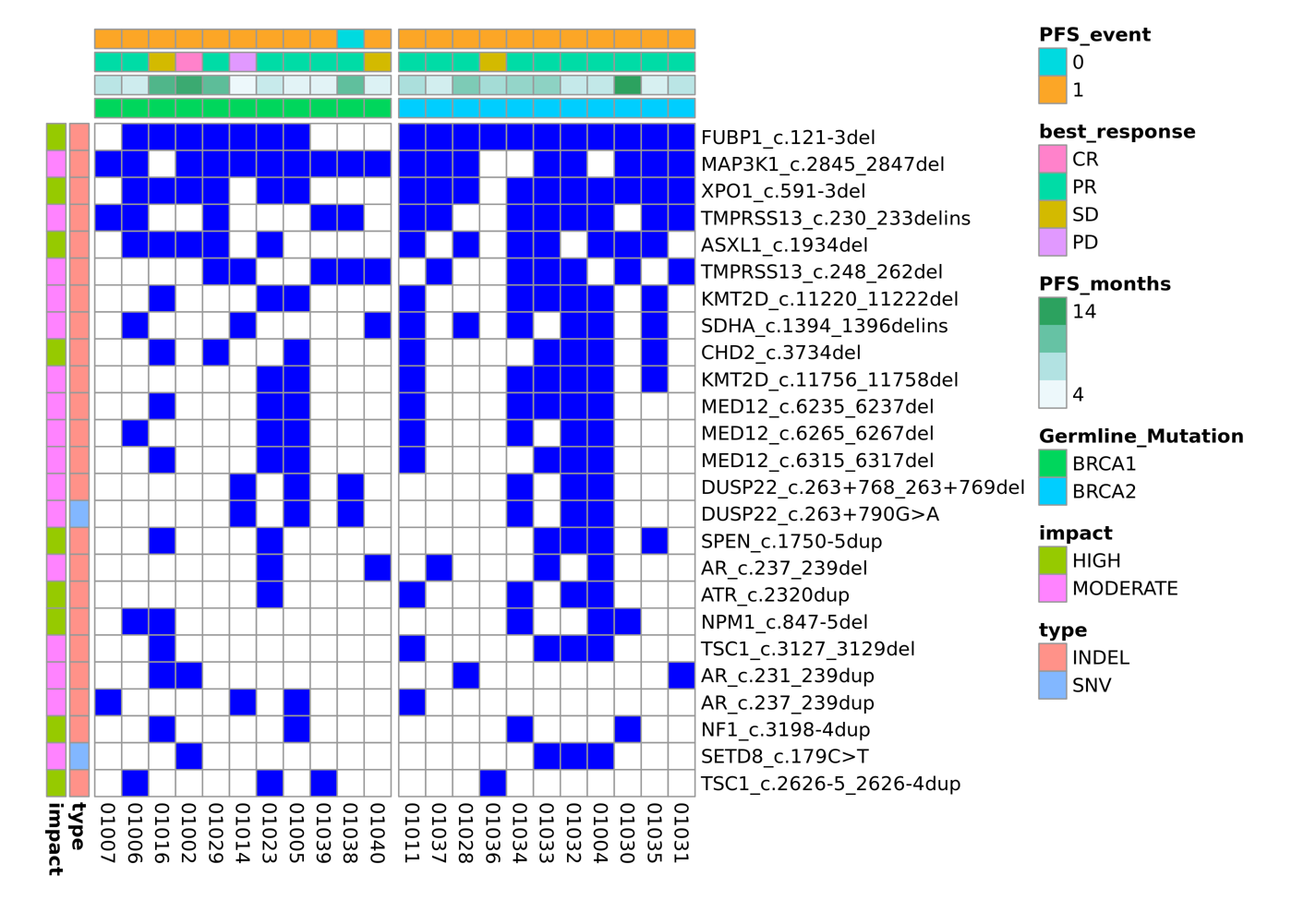


This heatmap illustrates the landscape of somatic mutations across patients in the main study cohort. CR, complete response; PD, disease progression; PR, partial response: SD, stable disease.

**Figure S2. Correlation between somatic mutations and treatment response (CR+PR) (at mutational level).**

**
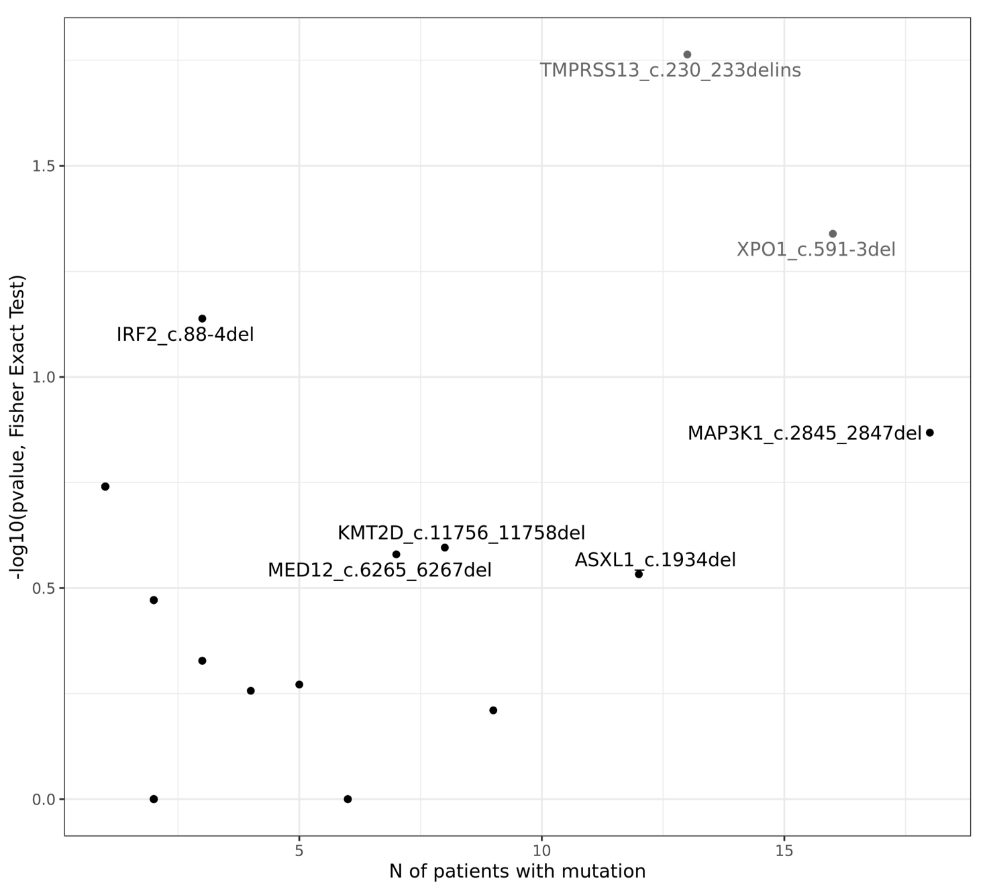
**

This plot illustrates the correlation between somatic mutations and treatment response among patients in the main study cohort, based on Fisher’s exact test.

**Figure S3. Prognostic significance of gene mutations in baseline and C2 samples.**

**
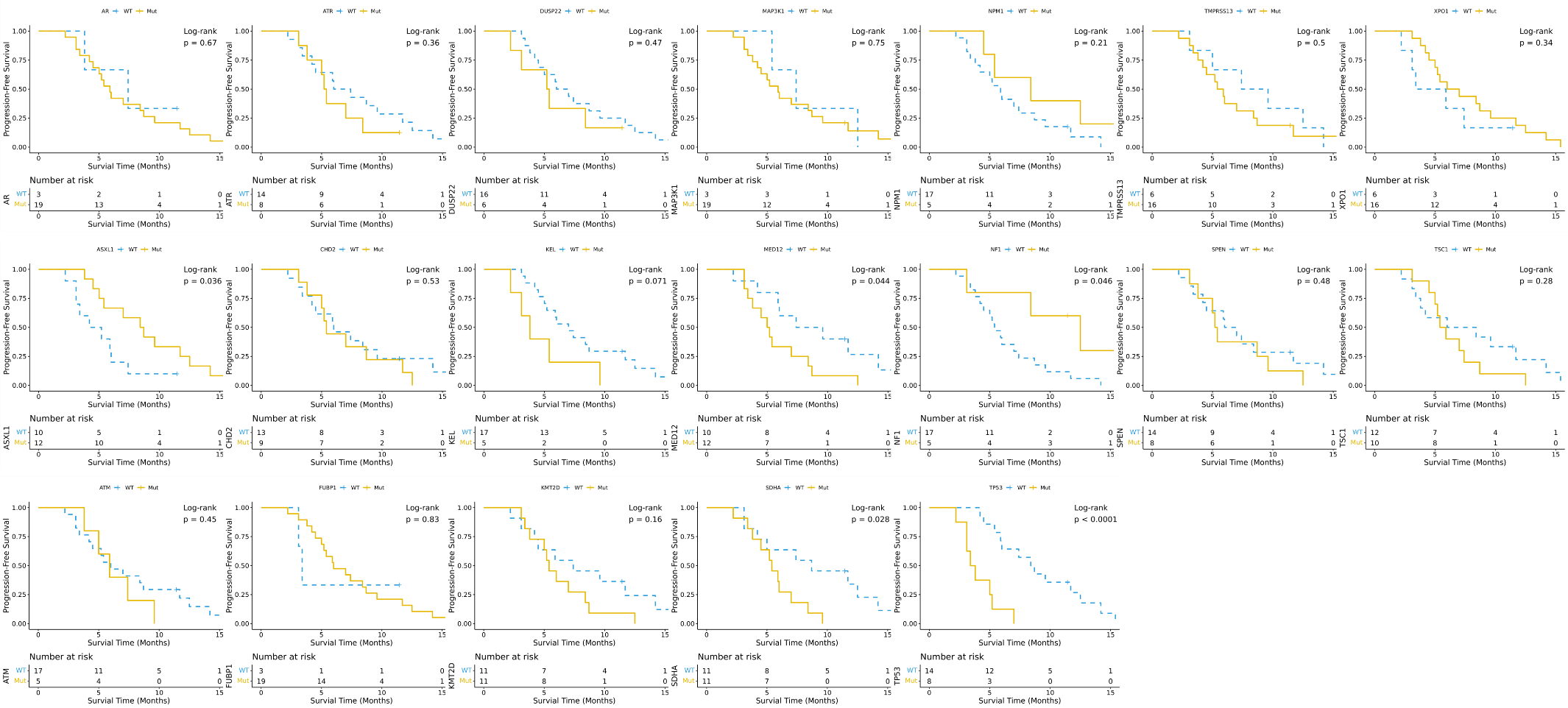
**

Kaplan-Meier curves for progression-free survival in the main study cohort stratified by 19 somatic genes with mutations in at least 5 patients.

**Table S1. Summary of prior therapies in the metastatic setting.**

| Patient ID | Cohort | (Neo)adjuvant therapy | Lines of chemotherapy in metastatic setting | Lines of endocrine therapy in metastatic setting |
| --- | --- | --- | --- | --- |
| 01002 | Main cohort | FEC-T | 1 (no platinum) | 0 |
| 01003 | Main cohort | wPTX | 1 (no platinum) | 0 |
| 01004 | Main cohort | TEC | 0 | 0 |
| 01005 | Main cohort | AC-T | 2 (carboplatin) | 0 |
| 01006 | Main cohort | / | 0 | 0 |
| 01007 | Main cohort | EC-T | 0 | 0 |
| 01010 | Main cohort | EC-T | 1 (no platinum) | 2 |
| 01011 | Main cohort | / | 3 (carboplatin, cisplatin） | 3 |
| 01014 | Main cohort | AC-T | 1 (no platinum) | 0 |
| 01016 | Main cohort | FAC-T | 2 (carboplatin) | 4 |
| 01018 | Main cohort | EC-T | 0 | 1 |
| 01019 | Main cohort | EC-T | 0 | 0 |
| 01020 | Main cohort | FEC-T | 0 | 0 |
| 01022 | Main cohort | EC-T | 1 (no platinum) | 0 |
| 01023 | Main cohort | AT*4 | 1 (cisplatin) | 0 |
| 01026 | Main cohort | EC-T | 1 (carboplatin) | 1 |
| 01028 | Main cohort | AC-T | 1 (no platinum) | 3 |
| 01029 | Main cohort | AC-T | 1 (no platinum) | 0 |
| 01030 | Main cohort | AC-T | 0 | 2 |
| 01031 | Main cohort | CEF | 1 (carboplatin) | 1 |
| 01032 | Main cohort | TE*6 | 2 (cisplatin) | 5 |
| 01033 | Main cohort | ddEC-T | 0 | 2 |
| 01034 | Main cohort | TC*6 | 3 (carboplatin) | 2 |
| 01035 | Main cohort | ET-ABX+Cb | 1 (carboplatin) | 0 |
| 01036 | Main cohort | / | 1 (carboplatin) | 3 |
| 01037 | Main cohort | EC-T | 0 | 2 |
| 01038 | Main cohort | EC-T | 1 (no platinum) | 0 |
| 01039 | Main cohort | ddEC-ddP | 0 | 0 |
| 01040 | Main cohort | / | 0 | 1 |
| 01027 | Exploratory cohort | / | 1 (no platinum) | 0 |
| 01001 | Exploratory cohort | EC-T | 2 (cisplatin) | 0 |
| 01012 | Exploratory cohort | AC-T*2, ABX+Cb*2 | 2 (carboplatin) | 0 |
| 01013 | Exploratory cohort | / | 1 (no platinum) | 0 |
| 01015 | Exploratory cohort | / | 3 (no platinum) | 0 |
| 01025 | Exploratory cohort | / | 1 (carboplatin) | 0 |
| 01009 | Exploratory cohort | FEC*5-TEC*1 | 1 (carboplatin) | 3 |
| 01017 | Exploratory cohort | ECF*6 | 1 (no platinum) | 3 |

**Table S2. Subgroup analyses of efficacy by clinical features.**

| Characteristics | ORR, No. (%), p-value* | | |  | DCR, No. (%), p-value* | | |  | Median PFS, months (95% CI), p-value** | |
| --- | --- | --- | --- | --- | --- | --- | --- | --- | --- | --- |
| Age, years, mean (range) |  | | 0.410 |  |  | | 1.000 |  |  | 0.548 |
| <45 | 11 | (69) |  |  | 15 | (94) |  |  | 7.0 (3.3-10.7) |  |
| ≥45 | 11 | (85) |  |  | 13 | (100) |  |  | 8.4 (5.3-11.5) |  |
| Subtype |  |  | 0.080 |  |  |  | 0.483 |  |  | 0.409 |
| HR+/HER2- | 9 | (60) |  |  | 15 | (100) |  |  | 7.3 (5.5-9.1) |  |
| TNBC | 13 | (93) |  |  | 13 | (93) |  |  | 5.9 (NA-12.1) |  |
| Visceral metastases |  |  | 1.000 |  |  |  | 0.172 |  |  | 0.196 |
| Yes | 18 | (75) |  |  | 24 | (100) |  |  | 7.0 (4.8-9.2) |  |
| No | 4 | (80) |  |  | 4 | (80) |  |  | 8.4 (3.0-13.8) |  |
| Prior chemotherapy in metastatic setting |  |  | 1.000 |  |  |  | 1.000 |  |  | 0.373 |
| ≤1 line | 18 | (75) |  |  | 23 | (96) |  |  | 7.3 (4.2-10.4) |  |
| ≥2 lines | 4 | (80) |  |  | 5 | (100) |  |  | 7.0 (3.1-10.9) |  |
| Prior platinum |  |  | 0.665 |  |  |  | 1.000 |  |  | 0.266 |
| Yes | 7 | (70) |  |  | 10 | (100) |  |  | 6.0 (3.2-8.8) |  |
| No | 15 | (79) |  |  | 18 | (95) |  |  | 8.5 (4.5-12.5) |  |
| Prior endocrine therapy in metastatic setting |  |  | **0.035** |  |  |  | 1.000 |  |  | 0.534 |
| Yes | 8 | (57) |  |  | 14 | (100) |  |  | 7.3 (6.6-8.0) |  |
| No | 14 | (93) |  |  | 14 | (93) |  |  | 5.9 (1.5-10.3) |  |

*P-value was determined by two-sided Fisher’s exact test.

**P-value was determined by Log-rank test.

ORR, objective response rate; DCR, disease control rate; PFS, progression-free survival; HR, hormone receptor; HER2, human epidermal growth factor receptor 2; TNBC, triple negative breast cancer.

**Table S3. Patient data on dose reductions and treatment interruptions due to toxicity.**

| Patient | Cohort | Mutation | Dose (HX008+Niraparib) |
| --- | --- | --- | --- |
| 01001 | Exploratory cohort3 | BRCA1 | 200mg/q3w+200mg/qd |
| 01002 | Main cohort | BRCA1 | 200mg/q3w+200mg/qd--niraparib reduce to 100mg/qd from C3 |
| 01003 | Main cohort | BRCA1 | 200mg/q3w+200mg/qd--niraparib reduce to 100mg/qd from C9 |
| 01004 | Main cohort | BRCA2 | 200mg/q3w+200mg/qd--niraparib reduce to 100mg/qd from C2 |
| 01005 | Main cohort | BRCA1 | 200mg/q3w+200mg/qd--niraparib reduce to 100mg/qd from C2 |
| 01006 | Main cohort | BRCA1 | 200mg/q3w+200mg/qd |
| 01007 | Main cohort | BRCA1 | 200mg/q3w+200mg/qd--niraparib reduce to 100mg/qd from C4 |
| 01009 | Exploratory cohort1 | CHEK2 | 200mg/q3w+200mg/qd |
| 01010 | Main cohort | PALB2 | 200mg/q3w+200mg/qd-niraparib reduce to 100mg/qd from C5 |
| 01011 | Main cohort | BRCA2 | 200mg/q3w+200mg/qd |
| 01012 | Exploratory cohort3 | BRCA1 | 200mg/q3w+200mg/qd--discontinued niraparib from C2 due to grade IV thrombocytopenia |
| 01013 | Exploratory cohort3 | PALB2 | 200mg/q3w+200mg/qd |
| 01014 | Main cohort | BRCA1 | 200mg/q3w+200mg/qd |
| 01015 | Exploratory cohort1 | CHEK2 | 200mg/q3w+200mg/qd--niraparib reduce to 100mg/qd from C7 |
| 01016 | Main cohort | BRCA1 | 200mg/q3w+200mg/qd--niraparib reduce to 100mg/qd from C4 |
| 01017 | Exploratory cohort1 | CHEK2 | 200mg/q3w+200mg/qd |
| 01018 | Main cohort | BRCA2 | 200mg/q3w+200mg/qd--niraparib reduce to 100mg/qd from C2 |
| 01019 | Main cohort | BRCA1 | 200mg/q3w+200mg/qd--niraparib reduce to 100mg/qd from C5 |
| 01020 | Main cohort | BRCA1 | 200mg/q3w+200mg/qd--niraparib reduce to 100mg/qd from C5 |
| 01022 | Main cohort | BRCA2 | 200mg/q3w+200mg/qd--niraparib reduce to 100mg/qd from C3 |
| 01023 | Main cohort | BRCA1 | 200mg/q3w+200mg/qd--niraparib reduce to 100mg/qd from C3 |
| 01025 | Exploratory cohort3 | BRCA2 | 200mg/q3w+200mg/qd--niraparib reduce to 100mg/qd from C4 |
| 01026 | Main cohort | BRCA1 | 200mg/q3w+200mg/qd |
| 01027 | Exploratory cohort2 | PALB2 | 200mg/q3w+200mg/qd--niraparib reduce to 100mg/qd from C3 |
| 01028 | Main cohort | BRCA2 | 200mg/q3w+200mg/qd |
| 01029 | Main cohort | BRCA1 | 200mg/q3w+200mg/qd |
| 01030 | Main cohort | BRCA2 | 200mg/q3w+200mg/qd |
| 01031 | Main cohort | BRCA2 | 200mg/q3w+200mg/qd |
| 01032 | Main cohort | BRCA2 | 200mg/q3w+200mg/qd--niraparib reduce to 100mg/qd from C2 |
| 01033 | Main cohort | BRCA2 | 200mg/q3w+200mg/qd |
| 01034 | Main cohort | BRCA2 | 200mg/q3w+200mg/qd--niraparib interrupted in C6 due to grade III anemia, resume medication from C7 (reduce to 100mg/qd) |
| 01035 | Main cohort | BRCA2 | 200mg/q3w+200mg/qd |
| 01036 | Main cohort | BRCA2 | 200mg/q3w+200mg/qd |
| 01037 | Main cohort | BRCA2 | 200mg/q3w+200mg/qd |
| 01038 | Main cohort | BRCA1 | 200mg/q3w+200mg/qd |
| 01039 | Main cohort | BRCA1 | 200mg/q3w+200mg/qd |
| 01040 | Main cohort | BRCA1 | 200mg/q3w+200mg/qd |

**Table S4. Change in mutations from baseline and C2 samples to PD samples.**

| **patient_ID** | **GERMLINEmut** | **best_response** | **PFS_months** | **PFS_event** | **type** | **gene_name** | **hgvs_c** | **hgvs_p** | **mutation_name** | **mutation_name.p** | **BLandC2** | **PD** | **change** |
| --- | --- | --- | --- | --- | --- | --- | --- | --- | --- | --- | --- | --- | --- |
| 01028 | BRCA2 | PR | 9.6 | 1 | INDEL | AR | c.1418_1420delGCG | p.Gly473del | AR_c.1418_1420del | AR_p.Gly473del | 0.3277 | 0.001 | -0.3267 |
| 01004 | BRCA2 | PR | 5.4 | 1 | INDEL | AR | c.1418_1420delGCG | p.Gly473del | AR_c.1418_1420del | AR_p.Gly473del | 0.444 | 0.001 | -0.443 |
| 01028 | BRCA2 | PR | 9.6 | 1 | INDEL | AR | c.231_239dupGCAGCAGCA | p.Gln78_Gln80dup | AR_c.231_239dup | AR_p.Gln78_Gln80dup | 0.358 | 0.001 | -0.357 |
| 01031 | BRCA2 | PR | 6 | 1 | INDEL | AR | c.231_239dupGCAGCAGCA | p.Gln78_Gln80dup | AR_c.231_239dup | AR_p.Gln78_Gln80dup | 0.3688 | 0.001 | -0.3678 |
| 01039 | BRCA1 | PR | 3.1 | 1 | INDEL | AR | c.234_239delGCAGCA | p.Gln79_Gln80del | AR_c.234_239del | AR_p.Gln79_Gln80del | 0.001 | 0.2425 | 0.2415 |
| 01033 | BRCA2 | PR | 8.7 | 1 | INDEL | AR | c.234_239delGCAGCA | p.Gln79_Gln80del | AR_c.234_239del | AR_p.Gln79_Gln80del | 0.2509 | 0.001 | -0.2499 |
| 01033 | BRCA2 | PR | 8.7 | 1 | INDEL | AR | c.237_239delGCA | p.Gln80del | AR_c.237_239del | AR_p.Gln80del | 0.2827 | 0.4403 | 0.1576 |
| 01004 | BRCA2 | PR | 5.4 | 1 | INDEL | AR | c.237_239delGCA | p.Gln80del | AR_c.237_239del | AR_p.Gln80del | 0.4383 | 0.001 | -0.4373 |
| 01014 | BRCA1 | PD | 2.2 | 1 | INDEL | DUSP22 | c.263+768_263+769delGT |  | DUSP22_c.263+768_263+769del | DUSP22_NA | 0.4131 | 0.001 | -0.4121 |
| 01005 | BRCA1 | PR | 3.1 | 1 | INDEL | DUSP22 | c.263+768_263+769delGT |  | DUSP22_c.263+768_263+769del | DUSP22_NA | 0.4409 | 0.001 | -0.4399 |
| 01032 | BRCA2 | PR | 5.2 | 1 | INDEL | DUSP22 | c.263+768_263+769delGT |  | DUSP22_c.263+768_263+769del | DUSP22_NA | 0.4735 | 0.001 | -0.4725 |
| 01014 | BRCA1 | PD | 2.2 | 1 | SNV | DUSP22 | c.263+790G>A |  | DUSP22_c.263+790G>A | DUSP22_NA | 0.4278 | 0.001 | -0.4268 |
| 01005 | BRCA1 | PR | 3.1 | 1 | SNV | DUSP22 | c.263+790G>A |  | DUSP22_c.263+790G>A | DUSP22_NA | 0.4702 | 0.001 | -0.4692 |
| 01032 | BRCA2 | PR | 5.2 | 1 | SNV | DUSP22 | c.263+790G>A |  | DUSP22_c.263+790G>A | DUSP22_NA | 0.5028 | 0.001 | -0.5018 |
| 01029 | BRCA1 | PR | 11.7 | 1 | INDEL | FUBP1 | c.121-3delT |  | FUBP1_c.121-3del | FUBP1_NA | 0.1347 | 0.001 | -0.1337 |
| 01028 | BRCA2 | PR | 9.6 | 1 | INDEL | FUBP1 | c.121-3delT |  | FUBP1_c.121-3del | FUBP1_NA | 0.1026 | 0.001 | -0.1016 |
| 01039 | BRCA1 | PR | 3.1 | 1 | INDEL | FUBP1 | c.121-3delT |  | FUBP1_c.121-3del | FUBP1_NA | 0.001 | 0.1557 | 0.1547 |
| 01033 | BRCA2 | PR | 8.7 | 1 | INDEL | FUBP1 | c.121-3delT |  | FUBP1_c.121-3del | FUBP1_NA | 0.1338 | 0.001 | -0.1328 |
| 01035 | BRCA2 | PR | 3.8 | 1 | INDEL | FUBP1 | c.121-3delT |  | FUBP1_c.121-3del | FUBP1_NA | 0.1385 | 0.001 | -0.1375 |
| 01031 | BRCA2 | PR | 6 | 1 | INDEL | FUBP1 | c.121-3delT |  | FUBP1_c.121-3del | FUBP1_NA | 0.1227 | 0.2338 | 0.1111 |
| 01007 | BRCA1 | PR | 5.9 | 1 | INDEL | NF1 | c.3198-4dupT |  | NF1_c.3198-4dup | NF1_NA | 0.001 | 0.3129 | 0.3119 |
| 01036 | BRCA2 | SD | 7.4 | 1 | INDEL | NF1 | c.3198-4dupT |  | NF1_c.3198-4dup | NF1_NA | 0.001 | 0.3397 | 0.3387 |
| 01032 | BRCA2 | PR | 5.2 | 1 | INDEL | NF1 | c.3198-4dupT |  | NF1_c.3198-4dup | NF1_NA | 0.001 | 0.2857 | 0.2847 |
| 01028 | BRCA2 | PR | 9.6 | 1 | SNV | SOD2 | c.81C>A | p.Ser27Arg | SOD2_c.81C>A | SOD2_p.Ser27Arg | 0.4277 | 0.5568 | 0.1291 |
| 01004 | BRCA2 | PR | 5.4 | 1 | SNV | SOD2 | c.81C>A | p.Ser27Arg | SOD2_c.81C>A | SOD2_p.Ser27Arg | 0.6028 | 0.7378 | 0.135 |
| 01029 | BRCA1 | PR | 11.7 | 1 | INDEL | TMPRSS13 | c.248_262delAGGCATCTCCAGCCC | p.Gln83_Ala87del | TMPRSS13_c.248_262del | TMPRSS13_p.Gln83_Ala87del | 0.6866 | 0.001 | -0.6856 |
| 01014 | BRCA1 | PD | 2.2 | 1 | INDEL | TMPRSS13 | c.248_262delAGGCATCTCCAGCCC | p.Gln83_Ala87del | TMPRSS13_c.248_262del | TMPRSS13_p.Gln83_Ala87del | 0.5371 | 0.4018 | -0.1353 |
| 01032 | BRCA2 | PR | 5.2 | 1 | INDEL | TMPRSS13 | c.248_262delAGGCATCTCCAGCCC | p.Gln83_Ala87del | TMPRSS13_c.248_262del | TMPRSS13_p.Gln83_Ala87del | 0.7052 | 0.001 | -0.7042 |
| 01035 | BRCA2 | PR | 3.8 | 1 | INDEL | TMPRSS13 | c.248_262delAGGCATCTCCAGCCC | p.Gln83_Ala87del | TMPRSS13_c.248_262del | TMPRSS13_p.Gln83_Ala87del | 0.001 | 0.8721 | 0.8711 |
| 01031 | BRCA2 | PR | 6 | 1 | INDEL | TMPRSS13 | c.248_262delAGGCATCTCCAGCCC | p.Gln83_Ala87del | TMPRSS13_c.248_262del | TMPRSS13_p.Gln83_Ala87del | 0.753 | 0.001 | -0.752 |
| 01029 | BRCA1 | PR | 11.7 | 1 | INDEL | XPO1 | c.591-3delT |  | XPO1_c.591-3del | XPO1_NA | 0.1335 | 0.001 | -0.1325 |
| 01039 | BRCA1 | PR | 3.1 | 1 | INDEL | XPO1 | c.591-3delT |  | XPO1_c.591-3del | XPO1_NA | 0.001 | 0.1238 | 0.1228 |
| 01033 | BRCA2 | PR | 8.7 | 1 | INDEL | XPO1 | c.591-3delT |  | XPO1_c.591-3del | XPO1_NA | 0.1079 | 0.001 | -0.1069 |
| 01031 | BRCA2 | PR | 6 | 1 | INDEL | XPO1 | c.591-3delT |  | XPO1_c.591-3del | XPO1_NA | 0.1106 | 0.001 | -0.1096 |

**Table S5. Post-protocol treatment for the main cohort.**

| Patient ID | First-line treatment after PD | Second-line treatment after PD | Third-line treatment after PD | Fourth-line treatment after PD | Fifth-line treatment after PD | Sixth-line treatment after PD |
| --- | --- | --- | --- | --- | --- | --- |
| 01002 | NA |  |  |  |  |  |
| 01003 | NA |  |  |  |  |  |
| 01004 | nab-paclitaxel+carboplatin+bevacizumab | capecitabine | letrozole+everolimus | nab-paclitaxel | vinorelbine |  |
| 01005 | gemcitabine+cisplatin+bevacizumab | vinorelbine+bevacizumab |  |  |  |  |
| 01006 | NA |  |  |  |  |  |
| 01007 | nab-paclitaxel+cisplatin |  |  |  |  |  |
| 01010 | nab-paclitaxel+carboplatin+bevacizumab |  |  |  |  |  |
| 01011 | NA |  |  |  |  |  |
| 01014 | NA |  |  |  |  |  |
| 01016 | abemaciclib+letrozole | BL-B01D1 | UTD1+bevacizumab |  |  |  |
| 01018 | nab-paclitaxel+carboplatin |  |  |  |  |  |
| 01019 | NA |  |  |  |  |  |
| 01020 | docetaxel+carboplatin |  |  |  |  |  |
| 01022 | nab-paclitaxel+carboplatin+bevacizumab |  |  |  |  |  |
| 01023 | NA |  |  |  |  |  |
| 01026 | JSKN003 |  |  |  |  |  |
| 01028 | nab-paclitaxel+carboplatin | capecitabine | IMP1734 |  |  |  |
| 01029 | NA |  |  |  |  |  |
| 01030 | DS8201 |  |  |  |  |  |
| 01031 | vinorelbine+capecitabine+bevacizumab | Etoposide |  |  |  |  |
| 01032 | RC48 | GQ1005 |  |  |  |  |
| 01033 | BL-B01D1 |  |  |  |  |  |
| 01034 | NA |  |  |  |  |  |
| 01035 | FDA018 |  |  |  |  |  |
| 01036 | BL-B01D1 | dalpiciclib+toremifene |  |  |  |  |
| 01037 | vinorelbine+capecitabine | UTD1+gemcitabine | chidamide+exemestane | nab-paclitaxel+carboplatin+bevacizumab | Etoposide | SKB264 |
| 01038 | NA |  |  |  |  |  |
| 01039 | nab-paclitaxel+carboplatin | eribulin+gemcitabine | vinorelbine+capecitabine |  |  |  |
| 01040 | NA |  |  |  |  |  |

PD, disease progression; NA, not available.
